# A case report on the ‘cobra twist’ technique: covering the posterior lobe of the left atrial appendage during percutaneous closure in a patient with challenging anatomy

**DOI:** 10.1093/ehjcr/ytae018

**Published:** 2024-01-25

**Authors:** Masanori Yamamoto, Ai Kagase, Ryo Yamaguchi, Tatsuya Tsunaki

**Affiliations:** Department of Cardiology, Toyohashi Heart Center, 21-1 Gobutori, Oyamachyo, Toyohashi, 441-8530 Aichi, Japan; Department of Cardiology, Nagoya Heart Center, 1-1-14, Sunadabashi, Higashi-ku, Nagoya, 461-0045 Aichi, Japan; Department of Cardiology, Gifu Heart Center, 4-14-4, Yabutaminami, Gifu, 500-8384 Gifu, Japan; Department of Cardiology, Nagoya Heart Center, 1-1-14, Sunadabashi, Higashi-ku, Nagoya, 461-0045 Aichi, Japan; Department of Cardiology, Toyohashi Heart Center, 21-1 Gobutori, Oyamachyo, Toyohashi, 441-8530 Aichi, Japan; Department of Cardiology, Toyohashi Heart Center, 21-1 Gobutori, Oyamachyo, Toyohashi, 441-8530 Aichi, Japan

An 84-year-old male with history of haemorrhagic stroke and paroxysmal atrial fibrillation under prescription of direct oral anticoagulation was admitted to our hospital. The predicted patient's stroke risk of CHA2DS2-VASc score was 5 and bleeding risk of HASBLED score was 5, respectively. Therefore, the left atrial appendage closure (LAAC) was indicated for this patient because of both high stroke and bleeding risk. Considering the peri-procedural transoesophageal echocardiography findings (*Figure 1A*), a 31 mm WATCHMAN-FLX (Boston Scientific, Marlborough, MA) device was chosen for LAAC. The double curve delivery sheath was inserted towards the LAA ostium inferiorly and posteriorly through the fossa ovale after trans-septal puncture. Although the device was released from the anterior lobe of the LAA (*Figure 1B* and *C*), more than half of the device was protruding, especially in the posterior lobe (*Figure 1D*). Multiple attempts of recapturing and re-deployment did not meet the device releasing criteria, causing the posterior lobe device protrusion. The main cause of this phenomenon was considered to be the misalignment of the delivery sheath and device orientation axes (*Figure 1E*). The inadequate collinearity worsened immediately after releasing the device. Therefore, we tried to forcibly rotate the sheath clockwise immediately before device deployment (see Supplementary material online, *Video*). This ‘cobra twist’ technique keeps the proximal WATCHMAN-FLX boll-like ‘cobra’ head in a manner that increases the working space (*Figure 1F*); thereafter, the delivery sheath axis was intentionally twisted clockwise at the timing of full device release (*Figure 1G*). After this manoeuvre, the WATCHMAN-FLX could be adequately aligned and implanted with acceptable device protrusion, including in the posterior lobe (*Figure 1H* and *I*). The issue of device protrusion in the posterior lobe is not a rare phenomenon during LAAC in cases of unfavourable device alignment. If conventional procedural approaches are ineffective, as in this case, the ‘cobra twist’ technique may be a treatment option to place the device into a deeper position in the posterior lobe.

**Figure 1:**
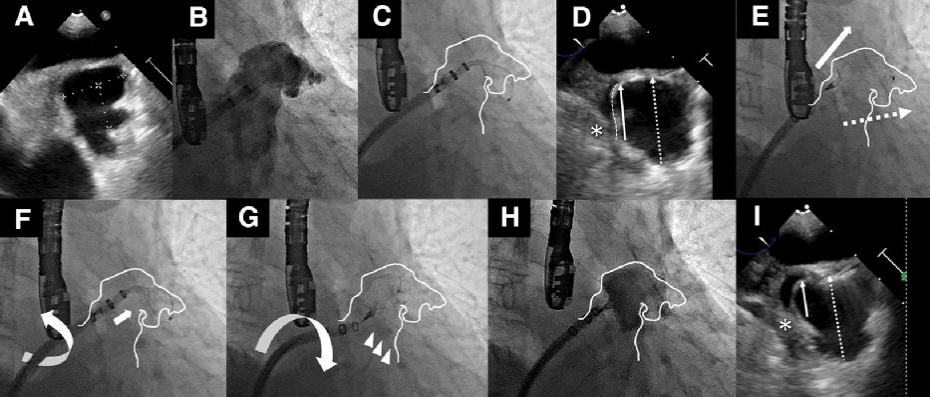
(*A*) Transoesophageal echocardiography (TEE) measurement of the maximum diameter of the left atrium appendage (LAA) orifice (25.0 mm); (*B*) baseline angiography; (*C*) the delivery sheath was placed in the anterior lobe, and the device was moved posteriorly; (*D*) in the TEE image, the extending device shoulder was represented by the white dot curve. TEE established the total length of device was calculated as 25.2 mm (white arrow dots), and the device's extension from the LAA ostium was measured at 16.9 mm (white arrow). Significant device protrusion of more than 50% from the LAA ostium was discovered along with a minor space (white asterisk) on the left side of device that did not meet the conventional device release criteria; (*E*) inadequate alignment between the delivery sheath (white arrow) and device (white arrow dots); video of the ‘cobra twist’ technique; (*F*) the delivery sheath was positioned using anticlockwise rotation (white arrow circle) and keeping the proximal device cobra-like head with forward pressure (white arrow); (*G*) the delivery sheath was then intentionally twisted clockwise (white circular arrow), which reduced the posterior device protrusion (white arrows); the process was deemed successful based on the results of (*H*) angiography and (*I*) TEE, which revealed that the device release criteria was <50% device protrusion from the LAA ostium [total device length: 25.5 mm (white arrow dots), protruded length: 12.0 mm (white arrow)] and no residual space in the left side of device (white asterisk).

## Supplementary Material

ytae018_Supplementary_Data

## Data Availability

Data sharing is not applicable to this report as there were no datasets.

## Supplementary material

**Consent:** Informed consent was obtained from the patient before the LAAC.

**Funding:** None.

